# Antigen Experience Shapes Phenotype and Function of Memory Th1 Cells

**DOI:** 10.1371/journal.pone.0065234

**Published:** 2013-06-07

**Authors:** Aaruni Khanolkar, Matthew A. Williams, John T. Harty

**Affiliations:** 1 Department of Microbiology, University of Iowa, Iowa City, Iowa, United States of America; 2 Department of Pathology, University of Utah, Salt Lake City, Utah, United States of America; 3 Interdisciplinary Graduate Program in Immunology, University of Iowa, Iowa City, Iowa, United States of America; 4 Department of Pathology, University of Iowa, Iowa City, Iowa, United States of America; New York University, United States of America

## Abstract

Primary and secondary (boosted) memory CD8 T cells exhibit differences in gene expression, phenotype and function. The impact of repeated antigen stimulations on memory CD4 T cells is largely unknown. To address this issue, we utilized LCMV and *Listeria monocytogenes* infection of mice to characterize primary and secondary antigen (Ag)-specific Th1 CD4 T cell responses. Ag-specific primary memory CD4 T cells display a CD62L^lo^CCR7^hi^ CD27^hi^ CD127^hi^ phenotype and are polyfunctional (most produce IFNγ, TNFα and IL-2). Following homologous prime-boost immunization we observed pathogen-specific differences in the rate of CD62L and CCR7 upregulation on memory CD4 T cells as well as in IL-2+IFNγco-production by secondary effectors. Phenotypic and functional plasticity of memory Th1 cells was observed following heterologous prime-boost immunization, wherein secondary memory CD4 T cells acquired phenotypic and functional characteristics dictated by the boosting agent rather than the primary immunizing agent. Our data also demonstrate that secondary memory Th1 cells accelerated neutralizing Ab formation in response to LCMV infection, suggesting enhanced capacity of this population to provide quality help for antibody production. Collectively these data have important implications for prime-boost vaccination strategies that seek to enhance protective immune responses mediated by Th1 CD4 T cell responses.

## Introduction

CD4 and CD8 T cells play a critical role in the host immune response to intracellular pathogens [1]–[4]. Following the initial exposure to the pathogen, T cells are primed, differentiate into effectors and undergo a phase of rapid expansion in numbers. This is followed by a sharp contraction phase in which 90–95% of the effector cells are culled, leaving behind a pool of Ag-experienced T cells that further differentiate into memory populations that can persist for long periods of time. Immunologic memory is a hallmark of the adaptive immune response and ensures the host of a swift response that efficiently eliminates the pathogen in the event of re-exposures [1]–[4]. The development of CD8 T cell memory has been examined in great detail in the past few years. For example, there is a general consensus that the initial CD8 T cells that survive the contraction phase express an effector-memory cell (Tem) phenotype, whereas memory CD8 T cell populations found long after clearance of infection are predominantly composed of central-memory T cells (Tcm) [2], [4], [5]. Tem and Tcm CD8 T cells subsets can be distinguished on the basis of expression of certain surface molecules and the secretion of IL-2. Classically, Tem express low levels of the homing receptors CD62L, CCR7 and produce low amounts of IL-2 while Tcm express higher levels of the CD62L and CCR7 and have a higher fraction of IL-2 producing cells [5]. Following a second exposure to the same pathogen the memory CD8 T cells develop into secondary effectors that eventually differentiate into secondary memory CD8 T cells. Secondary memory CD8 T cells maintain the Tem phenotype for extended time periods, and therefore differ from primary memory CD8 T cells that re-express CD62L more rapidly after priming [6]. This reacquisition of CD62L is also accompanied by improved IL-2 production [6], [7].

In contrast, CD4 T cell memory has not been as extensively studied and is complicated by the existence of multiple Th subsets [8]. Furthermore classification of CD4 T cell memory into Tem and Tcm subsets based primarily on CD62L expression is complicated by the failure of most memory CD4 T cells to re-express this lymph node homing receptor [9]–[11]. In addition, a substantial proportion of CD4 T cells produce IL-2 as early as 1 week after lymphocytic choriomeningitis virus (LCMV) and *Listeria monocytogenes* (Lm) infection and this property is retained as they transition into memory. This differs greatly from the almost complete absence of IL-2 production from effector CD8 T cells [6]. While some reports describe longitudinal analyses of primary and secondary Th1 memory cells [10], [12], [13], little is known about the functional differences induced by secondary immunization. Additionally it is unknown whether the qualities of secondary memory Th1 cells depend on the nature of the boosting agent, and this remains a key question in the development and evaluation of heterologous prime-boost vaccination strategies. In this study we have examined the hypothesis that memory Th1 cells demonstrate phenotypic and functional plasticity and repeat antigenic encounters induce functional maturation of memory Th1 cells. We analyzed both primary and secondary CD4 and CD8 T cell responses occurring simultaneously in the same host after both LCMV and Lm infections. Our data reveal that depending on the nature of the priming agent there are marked differences in the patterns of expression of CD62L, CCR7 and IL-2 production between CD4 and CD8 T cells, and some differences were also noted for a few of the markers between memory CD4 T cell populations generated by either LCMV or Lm. We also examined the impact of repeat antigenic encounters on the ability of memory CD4 T cell subsets to induce LCMV-specific neutralizing antibody (NAb) formation as a read out of helper function and observed a significant improvement in the kinetics of NAb generation in mice harboring secondary memory CD4 T cells.

## Materials and Methods

- Mice: C57BL/6 (B6) mice were obtained from the National Cancer Institute, Frederick, MD. B6.PL (Thy1.1^+^) and B6.SJL (CD45.1^+^) mice (Jackson Laboratories, Bar Harbor, ME) and Thy1.1^+^ SMARTA TCR transgenic mice [14] were maintained by brother-sister mating under SPF conditions until transfer to the appropriate biosafety level. Animals were maintained in accredited facilities at the University of Iowa (Iowa City, IA) and University of Utah (Salt Lake City, UT) and used in strict accordance with the recommendations in the Guide for the Care and Use of Laboratory Animals of the National Institutes of Health and all efforts were made to minimize suffering. The protocols were approved by the Institutional Animal Care and Use Committees (IACUC) at the University of Iowa (protocol number 12-01022) and University of Utah (protocol number 12-10011).
- Infections: The attenuated strain of *Listeria monocytogenes* (Lm) expressing ovalbumin, *actA^−^*LmOva, has been previously described [15]. Attenuated *actA^−^InlB^−^*Lm expressing the LCMV GP33/61 epitopes was obtained from Dr. Peter Lauer (Aduro Biotech, Berkeley, CA). For Lm infections, bacteria were grown and injected intravenously (i.v.) (1–1.5×10^7^ CFU/mouse) as previously described [15]. The number of colony forming units injected was confirmed by plating dilutions on selective media containing streptomycin (50 µg/ml). LCMV-Armstrong [2×10^5^ Plaque Forming Units (PFU)/mouse] was injected intraperitoneally (i.p.) as previously described [16].
- Antibodies and Reagents: The following antibodies conjugated with appropriate combination of fluorochromes were used in these studies: CD4; CD8α; CD45.1; Thy1.2; Thy1.1; IFN-γ; CD27; CD127; CD62L; TNFα, IL-2; IgG2a,κ; IgG2b; CCR7(eBiosciences); and BrdU; IgG1 (BD Pharmingen). In some experiments CCR7 expression was determined by using CCL19-human Fc fusion protein in combination with goat anti-human Fc-PE (eBiosciences). TAPI-2(HONHCOCH_2_CH(CH_2_CH(CH_3_)_2_)-CO-*t*-butyl-Gly-Ala-NHCH_2_CH_2_NH_2_) was purchased from Peptides International, Inc., (Louisville, KY) and used as described [6], [7]. BrdU incorporation was detected with the FITC-BrdU Flow Kit (BD Biosciences,San Jose, CA). Synthetic peptides GP33-41 (GP33), GP61-80 (GP61), OVA257-264 (OVA257), LLO190-201 (LLO190) have been described [17]–[20].
- Intracellular Cytokine Staining: Peptide stimulated intracellular cytokine staining was performed as previously described [16] using the cytofix/cytoperm kits from BD Pharmingen and Brefeldin A to enhance cytoplasmic cytokine detection. Splenocytes were treated with the TACE-inhibitor TAPI-2 to prevent cleavage of CD62L during stimulation as described [7]. Following the 5 h incubation, cells were washed and stained for cell surface and intracellular markers. Samples were subsequently acquired on a FACS-Calibur flow-cytometer (BD Biosciences). A minimum of 400,000 total events (greater at memory time points) were collected for each sample and analyzed using FlowJo software (Tree-Star Inc., Ashland, OR). Total numbers of antigen-specific CD4 and CD8 T cells were determined by multiplying the frequency of IFNγ+ cells with total numbers of CD4 or CD8 T cells in the spleen.
- BrdU-labeling experiments: BrdU was injected i.p. (2mg) on day 69 post LCMV infection and D55 post *actA^−^*LmOva infection and was also administered in drinking water (0.8mg/ml) for the next 7 days.
- Adoptive Transfer Experiments: A couple of days prior to the adoptive transfer total numbers and phenotypic and functional properties of antigen-specific CD4 and CD8 T cells were evaluated in the spleens of mice that had been previously infected with LCMV or *actA^−^*LmOva or a*ctA^−^InlB^−^*LmGP33/61. On the day of the adoptive transfer CD4 and CD8 T cells were enriched by negative selection using magnetic beads (Miltenyi-Biotec GmbH, Germany) from splenocytes obtained from donor mice and the indicated numbers of donor antigen-specific CD4 and CD8 memory T cells were transferred into naïve allelically-disparate recipients that were then infected the following day. Recipient mice received either a homologous or heterologous challenge one day after transfer.
- Plaque reduction neutralizing Ab titer (PRNT) assays: Primary and secondary memory SMARTA cells (Thy1.1^+^) were harvested from B6 (Thy1.2^+^) mice 60 days following infection with LCMV-Armstrong and 1×10^5^ of each subset was injected into separate groups of naïve B6 hosts. 5×10^3^ naïve SMARTA cells were transferred into an additional group of naïve B6 recipients. All recipient mice were infected with 2×10^5^ PFU of LCMV-Armstrong a day after the adoptive transfer. Serial serum samples were obtained from each recipient mouse beginning on day 8 post infection until day 120 post infection to assess the development of neutralizing Abs by performing plaque reduction neutralizing Ab titer (PRNT) assays adapted for LCMV based on previously described protocols [21], [22]. Two-fold dilutions of heat-inactivated serum samples (beginning at 1∶20 and ending at 1∶640) obtained from each infected and naïve control mouse were utilized in the PRNT assays. Additionally, pooled serum from immune mice (>180 days post LCMV-Arm infection) that did not receive SMARTA cells was also assayed at each time-point.
- Statistical analyses. Statistical analyses were performed using GraphPad software (GraphPad, San Diego, CA). Statistical significance was determined using an unpaired Student *t* test. A *p* value <0.05 was considered statistically significant.

## Results

### Primary CD4 T Cells Responses Following LCMV and Lm Infection

**Figure 1:**
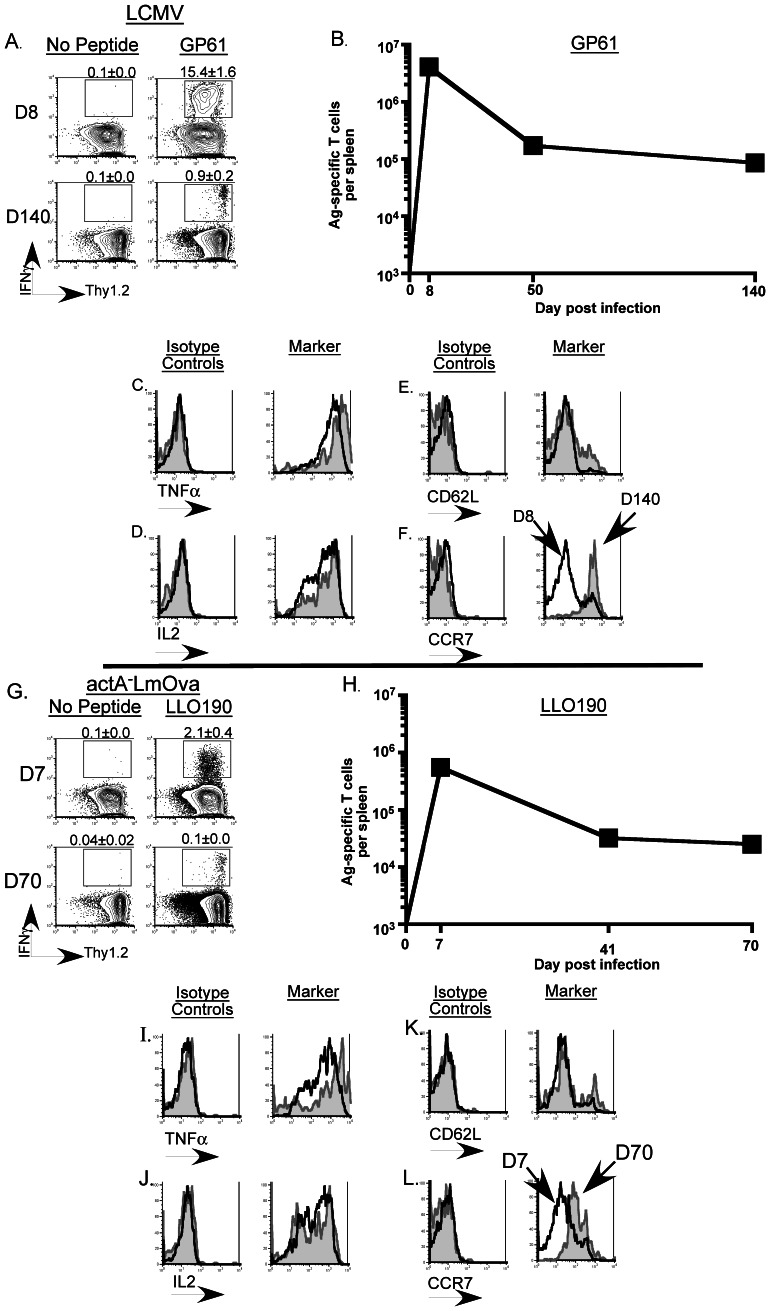
Examination of primary Ag-specific CD4 T cells responses in the spleens of infected mice. B6 mice were infected with LCMV-Armstrong (i.p.) or *actA^−^*LmOva (i.v.) and antigen-specific CD4 T cells responses were evaluated in the spleens at the indicated time-points following infection. (A) and (G) Representative contour-plots are gated on CD4 T cells and depict the frequency of the epitope-specific T cell responses (indicated by the numbers on top of each plot). (B) and (H) The line-graphs display the total numbers of antigen-specific CD4 T cells in the spleens of infected mice measured at the indicated time points. At least 3 mice per group were evaluated at each time point. (C–F) Representative histograms are gated on GP61 (LCMV)-specific (IFNγ^+^) CD4 T cells and (I–L) LLO190 (Lm)-specific (IFNγ^+^) CD4 T cells in the spleens and depict the expression patterns of the indicated molecules following staining with either the isotype-control Ab or the monoclonal Ab directed against the specific molecule. Each plot consists of the histogram depicting the expression pattern at the peak of the response following primary infection (black line) overlaid onto the corresponding expression pattern observed at the indicated memory time point (grey filled histogram).

Primary antigen-specific Th1 CD4 T cell responses were examined by peptide-stimulated intracellular cytokine staining for IFNγ at the indicated time-points in spleen cells from B6 mice infected with either LCMV-Armstrong or *actA^−^*LmOva (Fig. 1A–B; G–H). It has been reported that MHC-class II tetramer staining underestimates the true frequency of Ag-specific CD4 T cells [23] and in the setting of acute infections, as seen after LCMV-Armstrong and *actA^−^*LmOva exposure, intracellular cytokine staining offers the dual benefit of simultaneously assessing both the functional fitness and physical presence of the antigen-specific T cells [24]. Hence we utilized peptide-stimulated intracellular cytokine staining as the method of choice to examine Ag-specific T cell responses throughout this study. As previously described robust antigen-specific T cell responses for the relevant epitopes (Th1 responses to GP61-80 and LLO190-201 for LCMV and Lm CD4 responses, respectively, and GP33-41 and OVA257-264 for LCMV and Lm CD8 responses, respectively) were detected against both pathogens (Fig. 1 and Fig. S1) [20], [24], [25]. These responses were detected beyond 3 months post-LCMV infection and over 2 months post-Lm infection. While LCMV-specific memory populations can be easily tracked for over a year [24], the CD4 T cell memory responses in this Lm-infection model were difficult to measure reliably beyond D70 post-infection (LLO190 = 0.06% of CD4 T cells at D70 p.i.) (Fig. 1G). Additionally, memory progression occurs more rapidly following Lm infection versus LCMV infection [26]. Hence for subsequent experiments involving adoptive transfer of memory splenocytes, cells from Lm-infected mice were harvested at earlier time-points following establishment of memory as compared to LCMV-infected mice.

### Phenotypic and Functional Properties of Antigen-specific CD4 T Cells Following Primary Infection

Assessment of phenotype and function of LCMV and Lm-specific Th1 cells revealed that a majority of the effector (d7 or 8) Th1 CD4 T cells co-produced TNFα and IL-2 along with IFNγ and this property was retained as the cells transitioned into memory cells (Fig. 1C–D; I–J). Of note, while essentially all LCMV-specific memory CD4 T cells produce IL-2, Lm-specific Th1 CD4 T cells displayed a bimodal distribution of IL-2 co-producing cells that persisted into the memory phase (Fig. 1D; 1J). Consistent with recent literature [9]–[11], majority of LCMV and Lm-specific memory CD4 T cells did not re-acquire a CD62L^hi^ phenotype and while both LCMV and Lm-specific CD4 Th1 effector cells lost surface expression of CCR7, a chemokine receptor that similar to CD62L, is required for entry into lymph nodes, both populations upregulated CCR7 at similar rates as they developed into memory T cells (Fig. 1E–F; 1K–L). These data are in contrast to those observed with LCMV and Lm-specific CD8 T cells assayed in parallel. Effector CD8 T cells have a clearly defined subset of cells that do not co-produce IFNγ and TNFα and furthermore they do not produce IL2 (Fig. S1C–D; 1I–L). Additionally, only a small fraction of Ag-specific CD8 T cells acquire the ability to produce IL2 following establishment of memory (; 1J). Furthermore, CCR7 and CD62L expression patterns are concordant in CD8 T cells (Fig. S1E–F; 1K–L). Expression of CD27 and CD127 did not differ markedly between the antigen-specific CD4 and CD8 T cells following LCMV and Lm infection (data not shown). Collectively these data also demonstrate that IL-2 production and CD62L^hi^ expression are dissociated in memory CD4 T cell populations unlike their CD8 counterparts [6], [7]. Thus, primary memory differentiation of CD4 T cells exhibits substantial differences in cytokine production and expression of key trafficking molecules from CD8 memory T cell differentiation.

### Simultaneous Assessment of Primary and Secondary Antigen-specific CD4 T Cell Responses in the Same Host

**Figure 2:**
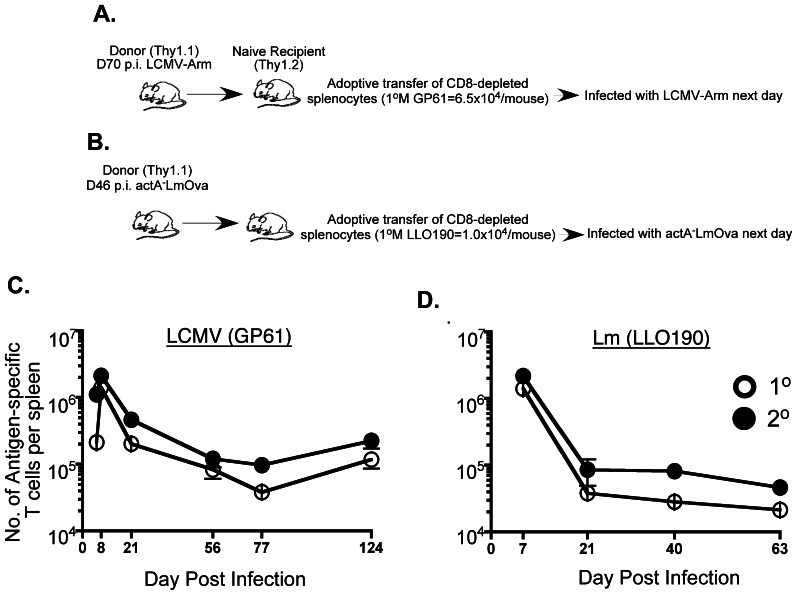
Kinetics of primary and secondary antigen-specific CD4 T cell responses following homologous prime-boost. Purified CD4 T cells derived from spleens of LCMV- and *actA^−^*LmOva-immune B6 donor (Thy1.1^+^) mice using negative selection were transferred into naïve recipients B6 (Thy1.2^+^) that were infected the following day as indicated (A–B). Total numbers of antigen-specific primary (endogenous: Thy1.1*^−^*IFNγ^+^) and secondary (transferred: Thy1.1^+^IFNγ^+^) responders were determined in the spleens of the recipients at the indicated time-points and are depicted in the line-graphs (C–D).

In an effort to control for potential differences in infection-induced inflammatory microenvironments that can develop and therefore influence primary and secondary T cell responses that are generated in separate hosts, naïve B6 (Thy1.2^+^) recipient mice were adoptively transferred with enriched CD4 T cells obtained from B6 (Thy1.1^+^) donors that had been previously infected with LCMV (day70 post-infection) (Fig. 2A) or *actA^−^*LmOva (day 46 post-infection) (Fig. 2B). Each CD4 T cell recipient received either 6.5 x 10^4^ Thy1.1^+^ GP61-specific or 1 × 10^4^ Thy1.1^+^ LLO190-specific primary memory CD4 T cells. Recipient mice were then given a homologous challenge the next day. This approach allowed us to visualize both the primary endogenous (Thy1.2^+^) and secondary transferred (Thy1.1^+^) responses simultaneously in the same host. Both the primary and secondary CD4 T cell responses peaked at day 8 post-LCMV infection, then contracted and formed memory populations that were easily detectable at 4 months post-infection (Fig. 2C). Both primary and secondary T cells responses showed robust expansion at day 7 post Lm-infection and could be detected up to 2 months post-infection (Fig. 2D). Primary and secondary CD8 T cell responses were analyzed in parallel in separate groups of mice (Fig. S2A–D). These results demonstrate that primary and secondary CD4 T cells behave similarly in terms of the kinetics of the response following a viral or bacterial infection when environmental parameters are equalized via adoptive transfer.

### Pathogen-specific Variation in Phenotypic Markers Between Primary and Secondary Antigen-specific Memory T Cells

One of the key observations of our previous study was the extended maintenance of the CD62L^lo^ phenotype on secondary memory CD8 T cells vis a vis the primary memory CD8 T cells [6], [27]. As expected, all of the antigen-specific T cells had reduced surface expression of CD62L measured at early time points after infection (data not shown). However, the extent of this CD62L downregulation varied between primary and secondary responders and also between LCMV and Lm-infected mice. Specifically, at the peak of the response 90% of LCMV and 77% of Lm-specific primary CD4 effectors downregulated CD62L and 96% of both LCMV and Lm-specific secondary CD4 effectors did the same. As for the CD8 T cells 97% of LCMV-specific and ∼80% of Lm-specific primary effector CD8 T cells downregulated CD62L, while over 96% of both LCMV and Lm-specific secondary CD8 effectors lost CD62L expression (data not shown).

**Figure 3:**
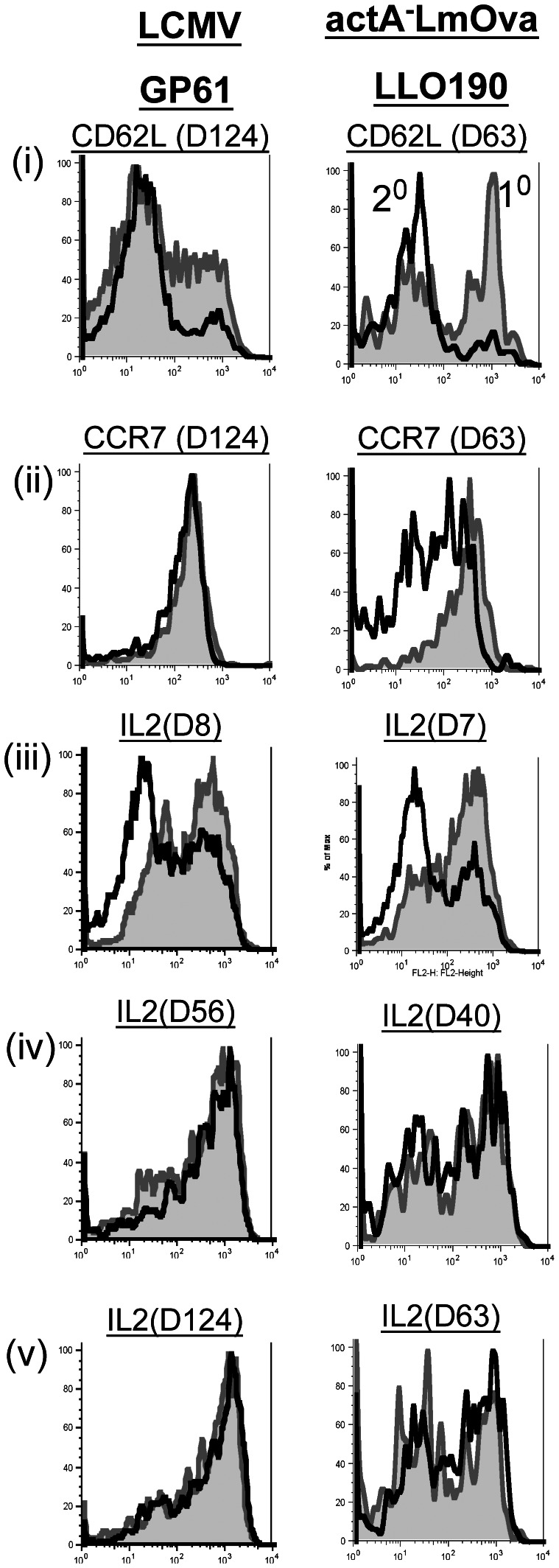
Assessment of CD62L, CCR7 expression and IL-2 production by antigen-specific CD4-T cells in homologous-prime-challenged mice. Antigen-specific T cells were identified by IFNγ staining in recipient mice as previously described. Representative histograms are gated on GP61 and LLO190-specific CD4 T cells T cells in the spleens and depict the expression patterns of the indicated molecule following antibody staining at the indicated time-points. Each plot consists of a histogram depicting the expression pattern of the relevant molecule on the primary (1°; endogenous) responders (grey filled histograms) and the secondary (2°; transferred) responders (black line).

Both primary and secondary Th1 CD4 T cell memory cells remained CD62L^lo^ even up to 124 days after LCMV infection while ∼50% of primary and <15% of secondary Lm-specific CD4 T cells had reacquired CD62L expression by day 63 post-infection [Fig. 3(i)]. CD62L upregulation on the primary memory Lm-specific CD4 T cell subset was more rapid in these homologous prime-challenged mice in comparison to LLO-190 specific primary memory CD4 T cells established in the absence of pre-existing memory CD4 T cells (Fig. 1K). CCR7 was similarly upregulated on LCMV-specific primary and secondary CD4 memory subsets while the kinetics of CCR7 upregulation were more protracted on Lm-specific secondary CD4 memory T cells [Fig. 3(ii)]. Consistent with our previous observations, the majority of the primary memory LCMV and Lm-specific CD8 T cells reacquired a CD62L^hi^ phenotype while only 25% of the LCMV-specific and less than 15% of the Lm-specific secondary memory CD8 T cells had upregulated CD62L at the same time point [Fig. S3(i)]. A similar trend was observed for CCR7 expression [Fig. S3(ii)]. Collectively, these results indicate intrinsic differences between CD4 and CD8 T cells and they again highlight the discordance between CD62L and CCR7 expression on antigen-specific memory CD4 T cells. Overall these data suggest the influence of both the pathogen and competition from pre-existing memory populations that share antigenic specificity with primary responders in regulating the expression of selected T cell markers.

### Most Secondary CD4 Effector T Cells do not Produce IL-2 in Homologous Prime-challenged Mice

An additional important distinction between memory CD8 T cell subsets is that secondary memory CD8 T cells exhibit a reduced proportion of IL-2 producing T cells [6], [7]. In contrast, while the fraction of IL-2-producing cells was markedly reduced in secondary Th1 CD4 T cell effectors after LCMV and Lm infection, this deficit was made up as these Th1 cells differentiated into memory [Fig. 3(iv–vi)]. Again, Lm elicited memory Th1 CD4 T cells displayed a bimodal pattern of IL-2 producers [Fig. 3(vi)]. IL-2 production by CD8 T cell memory subsets was similar to previous results [Fig. S3(vi)] [6]. Our findings highlight intrinsic differences between primary and secondary responders and between CD4 and CD8 T cells subsets in the temporal regulation of IL-2 production.

### Primary and Secondary Memory CD4 T Cells Display Similar Basal Proliferation Rates

**Figure 4:**
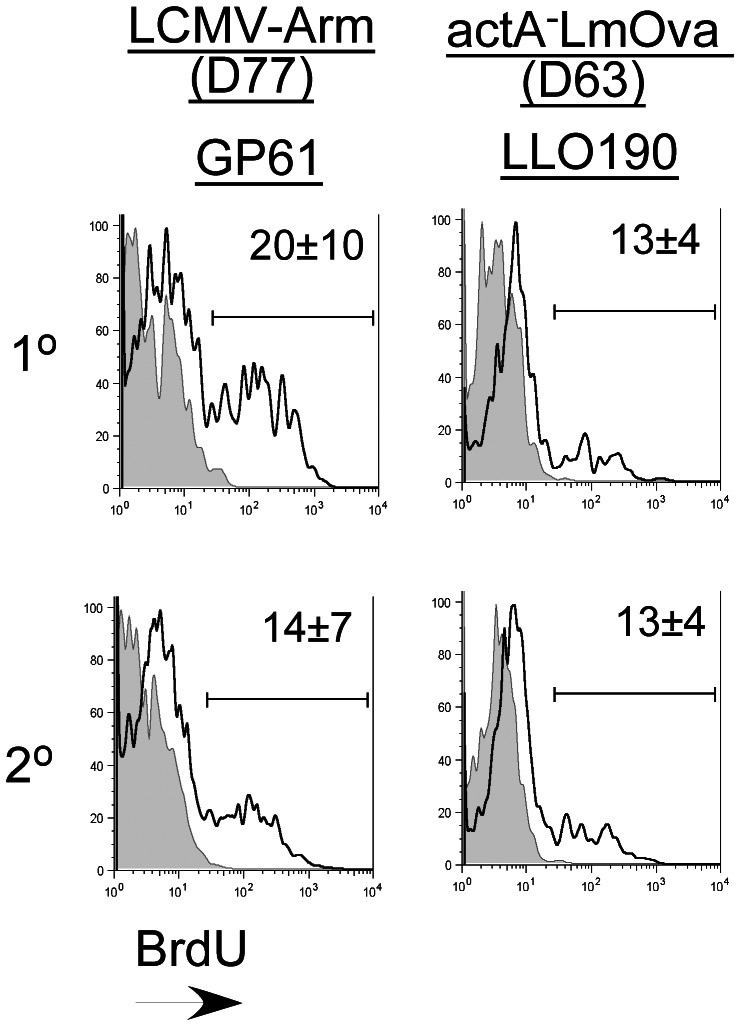
Homeostatic proliferation rates of primary (1°) and secondary (2°) antigen-specific memory CD4-T cells are similar. Homeostatic proliferation was assessed based on BrdU incorporation by antigen-specific 1° and 2° antigen-specific CD4 T cells evaluated simultaneously in the same host at the indicated memory time-points following LCMV and *actA^−^*LmOva infection. Each histogram plot is gated on GP61 or LLO190-specific CD4 T cells identified in the spleens and shows the fraction of BrdU^+^ cells (black line). Isotype control staining is depicted by the shaded grey histograms. Data are representative of results obtained from 3 mice analyzed per group.

We previously demonstrated that primary memory CD8 T cells display a higher basal turnover rate as compared to secondary memory CD8 T cells [6], [28]. These data were based on BrdU-incorporation studies performed in naïve mice adoptively transferred separately with either naïve OT-1 or primary memory OT-1 T cells that were subsequently challenged with *actA^−^*LmOva [6], [28]. Here we evaluated the basal turnover rates of primary and secondary non-transgenic memory CD4 T cells resident in the same host following a homologous challenge. Similar to recently published observations [10] our analysis revealed that the basal proliferation rates of both primary and secondary CD4 memory subsets were similar following homologous prime-boost immunizations (Fig. 4). Interestingly, BrdU incorporation by CD8 memory subsets examined in parallel was also not significantly different between the two memory subsets, although the homeostatic proliferation rate was slightly higher in the primary memory CD8 T cell subset (data not shown). This result is slightly different from our previous observations using transgenic OT-1 T cells and could be a result of the difference in experimental set up described above and might reflect competition for resources between the two memory subsets existing in the same host to maintain homeostatic proliferation [6], [28].

### Selective Modulation of Phenotypic and Functional Properties of Antigen-specific CD4 T Cells Following Heterologous Prime-challenge

**Figure 5:**
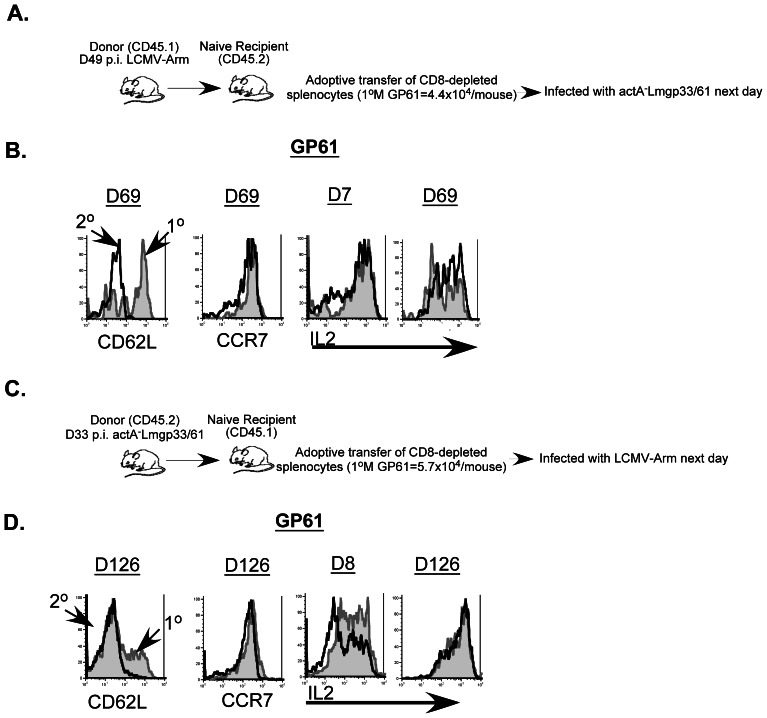
CD62L and IL-2 expression-patterns are selectively altered on memory CD4-T cell subsets following a heterologous-prime-challenge. (A). Bulk CD4 T cells were purified from spleens of LCMV-infected donor B6 (CD45.1^+^) mice using negative selection at the indicated time-point after infection and transferred into naïve B6 recipients (CD45.2^+^) that were challenged the following day with *actA^−^*LmGP33/61. (B) Surface expression of CD62L, CCR7 and elaboration of IL-2 was evaluated on primary (endogenous) responders (grey filled histograms) and secondary (transferred) responders (black line). In the converse heterologous prime-challenge experiment, bulk CD4 T cells were purified from spleens of *actA^−^*LmGP33/61-infected donor B6 (CD45.2^+^) mice using negative selection at the indicated time-point after infection and transferred into naïve B6 recipients (CD45.1^+^) that were challenged the following day with LCMV-Arm (C). (D) Surface expression of CD62L, CCR7 and elaboration of IL-2 was evaluated on primary (endogenous) responders (grey filled histograms) and secondary (transferred) responders (black line).

The homologous prime-challenge experiments examining primary and secondary T cell responses revealed some differences in the expression patterns of CD62L, CCR7 and IL-2 depending on whether LCMV or *actA^−^*LmOva was used as the infectious agent (Fig. 3). To determine if these characteristics are permanently imprinted after priming, naive B6 (CD45.2^+^) recipient mice were adoptively transferred with enriched CD4 T cells obtained from day 49 LCMV infected B6 (CD45.1^+^) donors and challenged a day later with *actA^−^*LmGP33/61 (Fig. 5A). Expression of CD62L and IL-2 was altered in mice that received the heterologous prime-challenge (Fig. 5B) compared to those that received an LCMV homologous prime-challenge (Fig. 3). Specifically, primary GP61-specific CD4 T cells upregulated CD62L at a faster rate than the secondary GP61-specific CD4 T cells. Additionally, secondary GP61-specific CD4 T cells did not display the marked reduction in the fraction of IL-2-producing cells at day 7 following *actA^−^*LmGP33/61 challenge (Fig. 5B). Furthermore, both primary and secondary GP61-specific CD4 T cell subsets displayed a bimodal pattern of IL-2 production at memory time points following the heterologous challenge, which contrasts with the LCMV homologous challenge model [Fig. 5B and Fig. 3(v–vi)]. Importantly, when the prime-challenge order was reversed (ie, Lm prime/LCMV challenge), both the CD62L and IL-2 phenotypes of primary and secondary memory displayed marked similarity to the LCMV homologous prime-challenge scenario (Fig. 5C–D and Fig. 3). Thus, in both scenarios the memory CD4 T cell response characteristics were dictated by the challenge infection rather than imprinted by priming.

### Mice Harboring Secondary Memory CD4 T Cells Display More Rapid Development of LCMV-specific Neutralizing Antibodies (NAb) Following Infection

**Figure 6:**
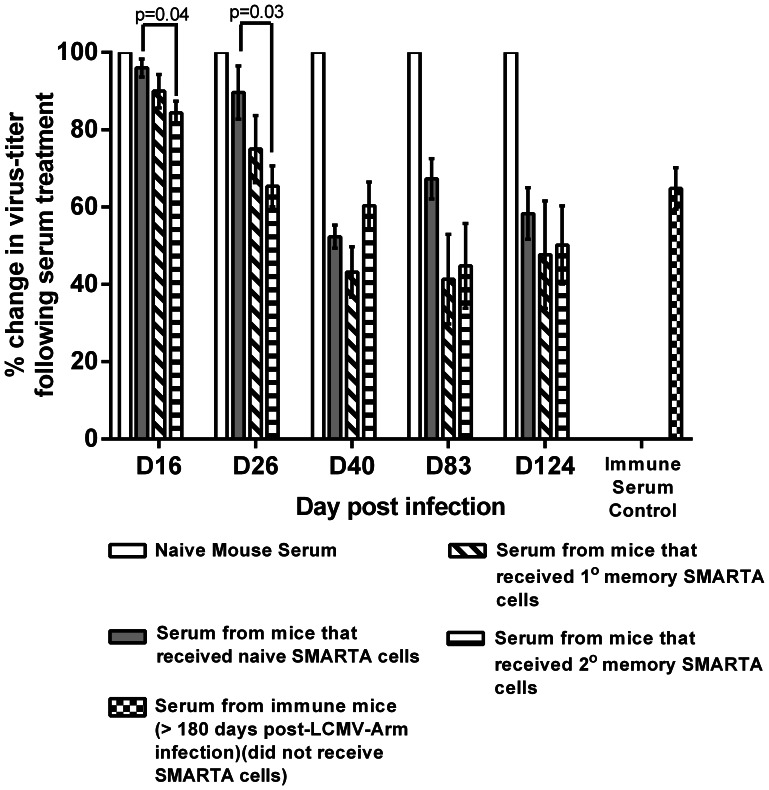
Virus-neutralization using sera obtained from LCMV-infected mice that received naïve, primary-(1°) or secondary-(2°) memory-SMARTA cells. Serum samples were obtained from each mouse at the indicated time points and analyzed by performing PRNT assays. The bar graphs depict the percent change in virus-titers following incubation of LCMV-Armstrong with a 1∶20 dilution of serum obtained from each mouse (n = 3–5 mice per group) compared with virus-titers measured following incubation of LCMV-Armstrong with 1∶20 dilution of serum obtained from naïve mice normalized to 100%. Data generated using pooled serum from immune mice (>180 days post-LCMV-Arm infection) that did not receive SMARTA cells and assayed at each time point was averaged for the entire data set and all data are expressed as mean±SEM and statistical significance was analyzed using the unpaired Student’s t test (p-value <0.05).

NAb comprise a critical component of protective immunity in a majority of vaccine-preventable diseases [21], [22]. It has been observed that the development of NAb is often delayed following infection with non-cytopathic viruses such as LCMV and HIV in comparison to cytopathic viruses such as poliovirus and VSV [29]–[35]. In the case of LCMV previous studies have demonstrated that this delayed and feeble NAb response (appearing variably between days 50–150 after primary infection) is attributable to a number of factors including the nature of the viral-glycoprotein and CD8 T cell-mediated destruction of infected B cells [30], [32]. Hence a number of these past studies have resorted to transient depletion of CD8 T cells prior to LCMV infection in an effort to boost NAb formation [29]–[35]. Additionally, it has been reported that LCMV-specific CD4 T cells recruited into a primary immune response are actively involved in delaying the development of a NAb response [33]. To address the effect prior antigenic experience would have on the ability of LCMV-specific CD4 T cells to influence NAb development, host mice that had received either naïve, primary memory or secondary memory SMARTA cells (specific for GP61–80) [14] were infected with LCMV and sera obtained from these mice were assayed for neutralizing activity in PRNT assays (Fig. 6). We observed a significant enhancement in LCMV-neutralizing activity in serum obtained from mice that received secondary memory SMARTA cells in comparison to mice that received naïve SMARTA cells as early as D16 following viral challenge. This was also observed at D26 post-infection but the advantage was lost by day 40 post-infection. Although mice that received primary memory SMARTA cells had lower virus titers than those that received naïve SMARTA cells at days 16 and 26 post-infection, the differences were not statistically significant. These data demonstrate that repeated antigenic exposure has the potential to enhance the helper function of memory CD4 T cells.

## Discussion

In this report we show that CD4 T cells exhibit both phenotypic and function alterations after repeated antigen encounter compared to CD8 T cells and that these changes are in part, reflective of the pathogen used for stimulation. Importantly, memory CD4 T cells exhibit selective plasticity of phenotypic and functional characteristics that reflect the boosting agent rather than the priming agent. Finally we show that a pre-existing secondary CD4 memory population transiently accelerates development of neutralizing LCMV-specific antibody development relative to naïve CD4 T cells.

One of the notable observations of our study is the discordance between CD62L and CCR7 expression on primary memory CD4 T cells in mice that have an intact endogenous immune repertoire. Specifically these cells displayed a CD62L^lo^ and CCR7^hi^ phenotype in contrast to primary memory CD8 T cells on which both markers were expressed at high levels. Previous studies have also demonstrated the maintenance of the CD62L^lo^ phenotype on endogenous memory CD4 T cells, although at least two studies have also demonstrated memory CD4 T cells that can reacquire high levels of CD62L [9]–[11], [36], [37]. However, in both of these latter studies CD62L expression was examined on transgenic memory CD4 T cells [36], [37]. As a result, the utility of CD62L in classifying CD4 memory T cells remains controversial, and a few recently published studies have utilized other markers, such as Ly6C, PSGL-1, T-bet and Blimp-1 to characterize memory CD4 T cells [36], [38]. Interestingly, in the *actA^−^*LmOva prime-challenged mice as well as in the heterologous prime-challenge experiment where *actA^−^*LmGP33/61 was used to challenge mice that received T cells from LCMV-immune donors, ≥ ∼ 50% of the primary memory GP61-specific CD4 T cells upregulated CD62L (47.5±2.4% in the homologous prime-challenge and 63.2±6.4% in the heterologous prime-challenge groups respectively), while over 90% of the secondary memory CD4 T cells maintained a predominantly CD62L^lo^ phenotype. By contrast, in both the homologous and heterologous prime-challenge experiment where LCMV was utilized as the challenge agent, 75% of the primary and greater than 85% of the secondary gp61-specific memory CD4 T cells failed to upregulate CD62L. The discordance observed in the extent of initial CD62L downregulation and subsequent rate of upregulation between primary and secondary responders could reflect the accelerated rate at which the secondary effectors (primary memory) are recruited into the immune response as compared to the primary effectors (naïve T cells). Additionally, the differences in the rate of reacquisition of a CD62L^hi^ phenotype between LCMV and Lm-infected hosts may reflect the replicative advantage of LCMV and its ability to induce a more vigorous immune response in comparison to attenuated Lm [39], [40].

In contrast to CD62L, CCR7 expression was reacquired at a much faster rate on memory CD4 T cells. Reacquisition of CCR7 in the face of reduced CD62L expression ensures continued access for CD4 memory T cells to the T cell zones of lymph nodes albeit via the afferent lymphatics rather than through blood vessels [41], and based on recently published reports, reacquisition of CCR7 suggests that most CD4 memory T cells in our study display some characteristics of a Tcm phenotype following LCMV and Lm infection [38]. Our data thus provides a cautionary note in the use of single markers to subset memory T cell populations.

Another interesting difference between CD4 and CD8 T cells is the proportion of cells that produce IL-2. Unlike the CD8 T cells, majority of Ag-specific CD4 T cells rapidly acquired the ability to co-produce IFNγ and IL-2 within a week following priming. This might reflect the status of CD4 T cells as the major source of IL-2 in the host and the role of IL-2 in supporting other critical immune subsets, such as CD8 T cells [42], [43]. Moreover, the ability to produce copious amounts of IL-2 early after priming also ensures optimal clonal expansion of naïve CD4 T cells by inducing miR-182 that suppresses Foxo1, an inhibitor of CD4 T cell proliferation [44]. Interestingly, in our homologous prime-challenge experiments as well as the heterologous prime-challenge study where LCMV was utilized as the challenge agent, the secondary effector CD4 T cells demonstrated a transient reduction in the proportion of cells co-producing IFNγ and IL-2. This diminution in the IFNγ-IL-2 co-producing subset has also been described in a recent publication where Tcm phenotype Th1 memory cells (CCR7^hi^T-bet^lo^CXCR5^+^ ) were deficient in their capacity to co-produce IFNγ and IL-2 in comparison to Tem phenotype Th1memory cells (CCR7^lo^T-bet^hi^ CXCR5*^−^*) [38]. This blunting of the IFNγ-IL-2 co-producing secondary-responder subset was less evident in the heterologous prime-challenge experiment where LCMV was used to prime and *actA^−^*LmGP33/61 was used to challenge. This could be due to differences in the relative distribution of Tcm and Tem in the transferred populations.

Our data also demonstrate that the presence of secondary memory Th1 cells in the host significantly accelerated the appearance of protective NAb. Hosts that harbored primary memory Th1 cells also demonstrated enhanced NAb production, albeit to a lesser degree. These data are noteworthy due to the fact that LCMV infection of mice represents a rather challenging model system to evaluate NAb responses. A general observation is that cytopathic viruses, such as polio, rabies and VSV are superior to their non-cytopathic counterparts, such as HIV and LCMV in generating NAb responses [29], [30], [32], [33]. One study attributed the feeble LCMV-specific NAb responses to an instrinsic property of the LCMV glycoprotein itself as infection of mice with recombinant LCMV expressing the VSV-glycoprotein generated robust and rapid NAb responses similar to the wild-type (WT)-VSV while infection of mice with recombinant VSV expressing the LCMV glycoprotein displayed delayed kinetics and very low titers of NAb responses similar to WT-LCMV [30]. Another study has implicated the robust LCMV-specific CD4 T cell responses elicited after LCMV infection as a contributor to the weak NAb responses by virtue of their ability to activate B cells of multiple specificities rather than concentrating their help towards stimulating B cells that are destined to generate NAb [33]. This link was examined by infecting mice with a strain of LCMV that lacks the GP61 epitope where both the kinetics and magnitude of NAb were enhanced [33]. Additionally, another study has demonstrated that one consequence of the robust Ag-specific CD8 T cell response to LCMV is the targeted destruction of infected B cells that generate NAb [32]. Hence a majority of previously published reports, including the two studies described above, have induced transient CD8 T cell depletion at the time of primary infection to demonstrate measurable LCMV-specific NAb responses [29]–[35]. In our present study we used mice with an intact endogenous T cell compartment to show that secondary memory CD4 T cells were more efficient in providing help for NAb production than both naïve and primary memory CD4 T cells. Our findings may reflect the possibility that secondary memory CD4 T cells generate more efficient Tfh responses that promote subsequent affinity maturation of Ab, and these possibilities are being currently investigated in our laboratory. Collectively these data demonstrate that the extent of prior antigenic experience can enhance helper function of memory CD4 T cells to improve protective humoral immune responses.

Overall our report has important implications for prime-boost vaccination strategies, as it provides evidence for the phenotypic plasticity of memory CD4 T cells that is shaped by the nature of the pathogen used to generate them. Additionally we provide proof of concept that multiple antigenic encounters can enhance the functional helper capacity of memory CD4 T cells.

## Supporting Information

Figure S1 **Examination of primary Ag-specific CD8 T cells responses in the spleens of infected mice.** B6 mice were infected with LCMV-Armstrong (i.p.) or *actA^−^*LmOva (i.v.) and antigen-specific CD8 T cells responses were evaluated in the spleens at the indicated time-points following infection. (A) and (G) Representative contour-plots are gated on CD8 T cells and depict the frequency of the epitope-specific T cell responses (indicated by the numbers on top of each plot). (B) and (H) The line-graphs display the total numbers of antigen-specific CD8 T cells in the spleens of infected mice measured at the indicated time points. At least 3 mice per group were evaluated at each time point. (C–F) Representative histograms are gated on GP33 (LCMV)-specific (IFNγ^+^) CD8 T cells and (I-L) OVA257 (Lm)-specific (IFNγ^+^) CD8 T cells in the spleens and depict the expression patterns of the indicated molecules following staining with either the isotype-control Ab or the monoclonal Ab directed against the specific molecule. Each plot consists of the histogram depicting the expression pattern at the peak of the response following primary infection (black line) overlaid onto the corresponding expression pattern observed at the indicated memory time point (grey filled histogram).
(TIFF)

Figure S2 **Kinetics of primary and secondary antigen-specific CD8 T cell responses following homologous prime-boost.** Purified CD8 T cells derived from spleens of LCMV- and *actA^−^*LmOva-immune B6 donor (Thy1.1^+^) mice using negative selection were transferred into naïve recipients B6 (Thy1.2^+^) that were infected the following day as indicated (A–B). Total numbers of antigen-specific primary (endogenous: Thy1.1*^−^*IFNγ^+^) and secondary (transferred: Thy1.1^+^IFNγ^+^) responders were determined in the spleens of the recipients at the indicated time-points and are depicted in the line-graphs (C–D).
(TIFF)

Figure S3 **Assessment of CD62L, CCR7 expression and IL-2 production by antigen-specific CD8-T cells in homologous-prime-challenged mice.** Antigen-specific T cells were identified by IFNγ staining in recipient mice as previously described. Representative histograms are gated on GP33 and OVA257-specific CD8 T cells T cells in the spleens and depict the expression patterns of the indicated molecule following antibody staining at the indicated time-points. Each plot consists of a histogram depicting the expression pattern of the relevant molecule on the primary (1°; endogenous) responders (grey filled histograms) and the secondary (2°; transferred) responders (black line).
(TIFF)
